# Illuminating Shared Genetic Associations Between Oesophageal Carcinoma and Pulmonary Carcinoma Risk

**DOI:** 10.7150/jca.92899

**Published:** 2024-03-04

**Authors:** Dengfeng Zhang, Jing Li, Tianxing Lu, Fangchao Zhao, Pengfei Guo, Zhirong Li, Xiaoliang Duan, Yishuai Li, Shujun Li, Jianhang Li

**Affiliations:** 1 Department of Thoracic Surgery, The Second Hospital of Hebei Medical University, Shijiazhuang, China.; 2 Department of Thoracic Surgery, Hebei Chest Hospital, Shijiazhuang, China.

**Keywords:** Lung cancer, Esophageal cancer, Pleiotropy, Genetic correlation, Genome-wide association study, Pleiotropic analysis under composite null hypothesis, Mendelian randomization

## Abstract

**Background:** Lung cancer and oesophageal cancer are prevalent malignancies with rising incidence and mortality worldwide. While some environmental and behavioural risk factors for these cancers are established, the contribution of genetic factors to their pathogenesis remains incompletely defined. This study aimed to interrogate the intricate genetic relationship between lung cancer and oesophageal cancer and their potential comorbidity.

**Methods:** We utilised linkage disequilibrium score regression (LDSC) to analyse the genetic correlation between oesophageal carcinoma and lung carcinoma. We then employed several approaches, including pleiotropic analysis under the composite null hypothesis (PLACO), multi-marker analysis of genomic annotation (MAGMA), cis-expression quantitative trait loci (eQTL) analysis, and a pan-cancer assessment to identify pleiotropic loci and genes. Finally, we performed bidirectional Mendelian randomisation (MR) to evaluate the causal relationship between these malignancies.

**Results:** LDSC revealed a significant genetic correlation between oesophageal carcinoma and lung carcinoma. Further analysis identified shared gene loci including PGBD1, ZNF323, and WNK1 using PLACO. MAGMA identified enriched pathways and 9 pleiotropic genes including HIST1H1B, HIST1H4L, and HIST1H2BL. eQTL analysis integrating oesophageal, lung, and blood tissues revealed 26 shared genes including TERT, NKAPL, RAD52, BTN3A2, GABBR1, CLPTM1L, and TRIM27. A pan-cancer exploration of the identified genes was undertaken. MR analysis showed no evidence for a bidirectional causal relationship between oesophageal carcinoma and lung carcinoma.

**Conclusions:** This study provides salient insights into the intricate genetic links between lung carcinoma and oesophageal carcinoma. Utilising multiple approaches for genetic correlation, locus and gene analysis, and causal assessment, we identify shared genetic susceptibilities and regulatory mechanisms. These findings reveal new leads and targets to further elucidate the genetic basis of lung and oesophageal carcinoma, aiding development of preventive and therapeutic strategies.

## 1. Introduction

Esophageal cancer, one of the most prevalent malignancies, has shown an alarming upward trend in recent years and is becoming more prevalent among younger populations. It ranks as the eighth most common cancer worldwide and the sixth leading cause of cancer-related deaths^1^, ^2^. Despite advancements in treatments like surgery combined with radiotherapy and chemotherapy, the prognosis for esophageal cancer patients remains generally poor. In most countries, the 5-year survival rate after diagnosis of esophageal cancer still ranges between 10% and 30%^3^. On the other hand, lung cancer stands as a primary cause of cancer-related mortality, accounting for over 20% of all cancer deaths^1^. Approximately 80% of lung cancer deaths are attributed to smoking, and the majority of patients present with signs of metastasis by the time symptoms manifest, which is a major factor contributing to the high mortality rate^4^, ^5^. Each year, millions of people are diagnosed with either esophageal cancer or lung cancer, and the death toll is also strikingly substantial^1^, ^6^. Despite significant progress in research on lung and esophageal cancers over the past decades, the exact mechanisms underlying their development remain incompletely understood.

Genetic factors play a crucial role in the development of cancer. Early familial studies have indicated a clustering of lung cancer and esophageal cancer within families, suggesting the potential importance of genetic factors in their susceptibility^7^-^10^. Previous research has found underlying mechanistic links between smoking status and the prognosis of lung and esophageal cancer patients, supporting the clinical use of mithramycin to inhibit ABCG2 and suppress tumor stem cell signaling^11^. Additionally, there are shared molecular pathophysiological factors between the two cancers that may promote tumor development, such as ECRG4, microRNA-93, ACTL6A, and FOXA1^12^-^15^. Furthermore, patients with esophageal cancer have an increased risk of developing lung cancer^16^, ^17^, and vice versa, patients with lung cancer face an elevated risk of esophageal cancer recurrence^18^. These associations suggest the possibility of common genetic or environmental risk factors between esophageal and lung cancers, an area that remains insufficiently explored. Therefore, a systematic analysis is necessary to elucidate whether there are shared pleiotropic risk variants between esophageal and lung cancers, and whether specific molecular pathways are involved in these interactions.

In recent years, Genome-Wide Association Studies (GWAS) have emerged as a powerful tool for unraveling the genetic basis of complex diseases^19^. The LDSC method is a robust technique used to assess the genetic correlation between diseases and complex traits^20^. This study will begin by utilizing the LDSC method, combined with extensive population-based GWAS data, to assess the genetic correlation between lung cancer and esophageal cancer. Subsequently, we will identify pleiotropic loci and genes using different approaches. In the PLACO analysis^21^, we will identify three shared gene loci: PGBD1, ZNF323, and WNK1. In the MAGMA analysis, we will uncover significantly enriched relevant pathways and further pinpoint nine pleiotropic genes, including HIST1H1B, HIST1H4L, and HIST1H2BL. Next, using data from esophageal tissues, lung tissues, and whole blood, we will identify eQTL genes associated with these pleiotropic risk loci, which will include 26 genes such as TERT, ZKSCAN3, NKAPL, ZNF165, RAD52, BTN3A2, GABBR1, HIST1H2BK, CLPTM1 and TRIM27. Subsequently, we will conduct a pan-cancer analysis on all the relevant genes obtained through these three methods. Finally, employing the MR analysis method, we will assess the causal relationship between esophageal cancer and lung cancer, and the results will demonstrate that there is no mutual causal relationship between the two types of cancer. Through this study's approach and methodology, we seek to understand the complex genetic interplay between lung cancer and esophageal cancer. It will lay a strong foundation for future research, driving new insights and goals in prevention, early diagnosis, and personalized treatments.

## 2. Methods

### 2.1 GWAS summary statistics

#### 2.1.1 Lung cancer data

The lung cancer data was derived from a GWAS meta-analysis^22^ that encompassed 14,803 cases and 12,262 controls from the European OncoArray consortium. The GWAS analysis was performed using a fixed-effects model (online methods) to combine OncoArray results with previously published lung cancer GWAS data, totaling 29,266 cases and 56,450 controls. In total, 18 susceptibility loci were identified with genome-wide significance, including 10 novel loci.

#### 2.1.2 Esophageal cancer data

The esophageal cancer data was collected through a GWAS meta-analysis^23^ conducted across four independent studies in Europe, North America, and Australia. All patients in the study had European ancestry and were confirmed to have the disease through histopathological examination. The meta-analysis employed a fixed-effects inverse-variance weighted method and included a total of 4,112 esophageal adenocarcinoma patients. Additionally, 17,159 representative controls were selected from four whole-genome association studies conducted in Europe, North America, and Australia.

### 2.2 TCGA statistics

The gene expression profiles and clinical data can be found at the GDC portal (https://portal.gdc.cancer.gov/). In order to gain insights into potential protein-protein interactions among the 34 genes, a protein-protein interaction (PPI) network was constructed using the Search Tool for the Retrueval of Interacting Genes (STRING) database.

### 2.3 Statistical analyses

#### 2.3.1 Genetic correlation analysis

In GWAS studies, both polygenicity and confounding factors (such as cryptic relatedness and population stratification) can lead to inflated test statistics. However, we cannot distinguish whether the inflation is due to polygenicity or confounding. Through linkage disequilibrium score regression (LDSC), we can quantify the contribution of each by studying the relationship between test statistics and linkage disequilibrium. Using the linkage disequilibrium score regression (LDSC)^24^ and high-definition likelihood (HDL)^25^ methods, we assessed the shared polygenic architecture among traits. The LD scores in LDSC were computed from European ancestry samples in the 1000 Genomes Project as the reference panel^26^. The reference set for HDL comprised 1,029,876 quality-controlled HapMap3 SNPs. We applied stringent quality control measures for SNP selection: (i) exclusion of non-biallelic SNPs and those with ambiguous strand information; (ii) removal of SNPs without rs tags; (iii) deletion of duplicate SNPs or those not present in the 1000 Genomes Project, as well as SNPs with mismatched alleles; (iv) exclusion of SNPs within the major histocompatibility complex region (chr6: 28.5-33.5Mb) due to its complex LD structure; (v) retention of SNPs with minor allele frequency (MAF) > 0.01.

#### 2.3.2 Pleiotropic analysis under composite null hypothesis (PLACO)

SNP-Level PLACO is a novel method that enables the study of pleiotropic loci between complex traits using only summary-level genotype-phenotype association statistics^21^, ^27^. To detect pleiotropic loci, we initially computed the square of the Z-scores for each variant and removed SNPs with extremely high Z^^2^ (>80). Additionally, considering the potential correlation between lung cancer and esophageal cancer, we estimated the correlation matrix of Z-scores. We then employed the Level α Intersecting Union Test (IUT) method to test the hypothesis of no pleiotropy. The final p-value from the IUT test represents the maximum p-value obtained from testing H0 against H1.

#### 2.3.3 Enrichment analysis for identified pleiotropic genes

Based on the PLACO results, we further mapped the identified loci to nearby genes to explore the shared biological mechanisms of these pleiotropic loci. We performed Generalized Gene-Set Analysis of GWAS Data (MAGMA) analysis^28^ on genes based on the PLACO output and single-trait GWAS at pleiotropic loci or overlapping with them.

MAGMA integrates gene information and pathway analysis of complex diseases in a biologically meaningful way, serving as an effective supplement to single-variant GWAS. This analysis aimed to identify candidate pathways and tissue enrichments for pleiotropic genes. Functional maps and annotations from the Functional Mapping and Annotation of Genome-Wide Association Studies (FUMA) were utilized to determine the biological functions of the pleiotropic loci^29^. Additionally, we employed FUMA for differential expression analysis and gene-set enrichment analysis of the pleiotropic genes identified by PLACO. To assess the biological significance of gene-set enrichment analysis, we investigated the detected pleiotropic genes against gene sets obtained from MsigDB (such as hallmark gene sets, positional gene sets, curated gene sets, motif gene sets, computational gene sets, and Gene Ontology sets), utilizing a series of pathway enrichment analyses based on the Molecular Signatures Database (MSigDB) to determine the functional roles of the mapped genes^30^. Incorporating SNP-gene association data, eQTL analysis was conducted, encompassing esophageal tissue, lung tissue, and whole-blood tissue.

#### 2.3.4 Mendelian randomization analysis

Mendelian randomization (MR) is a commonly used tool for causal inference, employing SNPs associated with the exposure to study its impact on the outcome^31^. It is important to highlight that MR not only provides insights into causal relationships between diseases but also offers meaningful genetic explanations for the nature of disease comorbidities by addressing horizontal or vertical pleiotropy issues^32^. To achieve this goal, we conducted bidirectional MR analyses as follows: We utilized the clumping program in PLINK software^33^ to select all independently associated significant genetic loci (P < 5×10^-8) with the disease as instrumental variables (IVs), with an r^2 threshold of 0.001 and a window set at 10,000kb. To ensure the strength of the IVs, we calculated the r^2 and F statistics for each instrumental variable^34^. The formula for the F statistic is as follows: F = (r^2 × (n - 2)) / (1 - r^2) where r^2 represents the proportion of variance explained by the instrumental variable, n is the sample size, and k is the number of SNPs. The primary method employed for Mendelian randomization is the inverse-variance weighted (IVW) method, which requires the IVs to satisfy three assumptions: (1) the IVs should be correlated with the exposure; (2) the IVs should not be associated with confounding factors related to both exposure and outcome; and (3) the IVs' effects on the outcome should be entirely mediated through the exposure. Several sensitivity analyses were conducted. Firstly, the IVW and MR-Egger's Q test were used to detect potential violations of the assumptions by assessing heterogeneity among individual IVs^35^. Secondly, we applied MR-Egger to estimate horizontal pleiotropy based on its intercept to ensure the genetic variation is independent of exposure and outcome^36^. Additional analyses using MR methods with different modeling assumptions and strengths (e.g., weighted median and weighted mode) were performed to enhance the stability and robustness of the results. All statistical analyses were conducted using R version 3.5.3, and MR analyses utilized the MendelianRandomization package^37^.

The weighted median method is a robust causal effect estimation approach that is based on the assumption that at least half of the instrumental variables are valid (i.e., not associated with confounders and only affect the outcome through the exposure of interest). This method uses the effect estimates and precision of each instrument (typically the inverse of the standard error) as weights to calculate the median. The weighted mode method is based on the modal estimate and calculates the causal effect estimate for each instrument. Using methods like kernel density estimation (KDE), it estimates the distribution of the estimates, taking into account the weights for each estimate, and finds the peak of that distribution, i.e., the modal value. The weighted mode method is less sensitive to the influence of individual invalid instruments, especially when these invalid instruments are not clustered around the same estimate value.

## 3. Results

### 3.1 Genetic correlation

**Figure 1:**
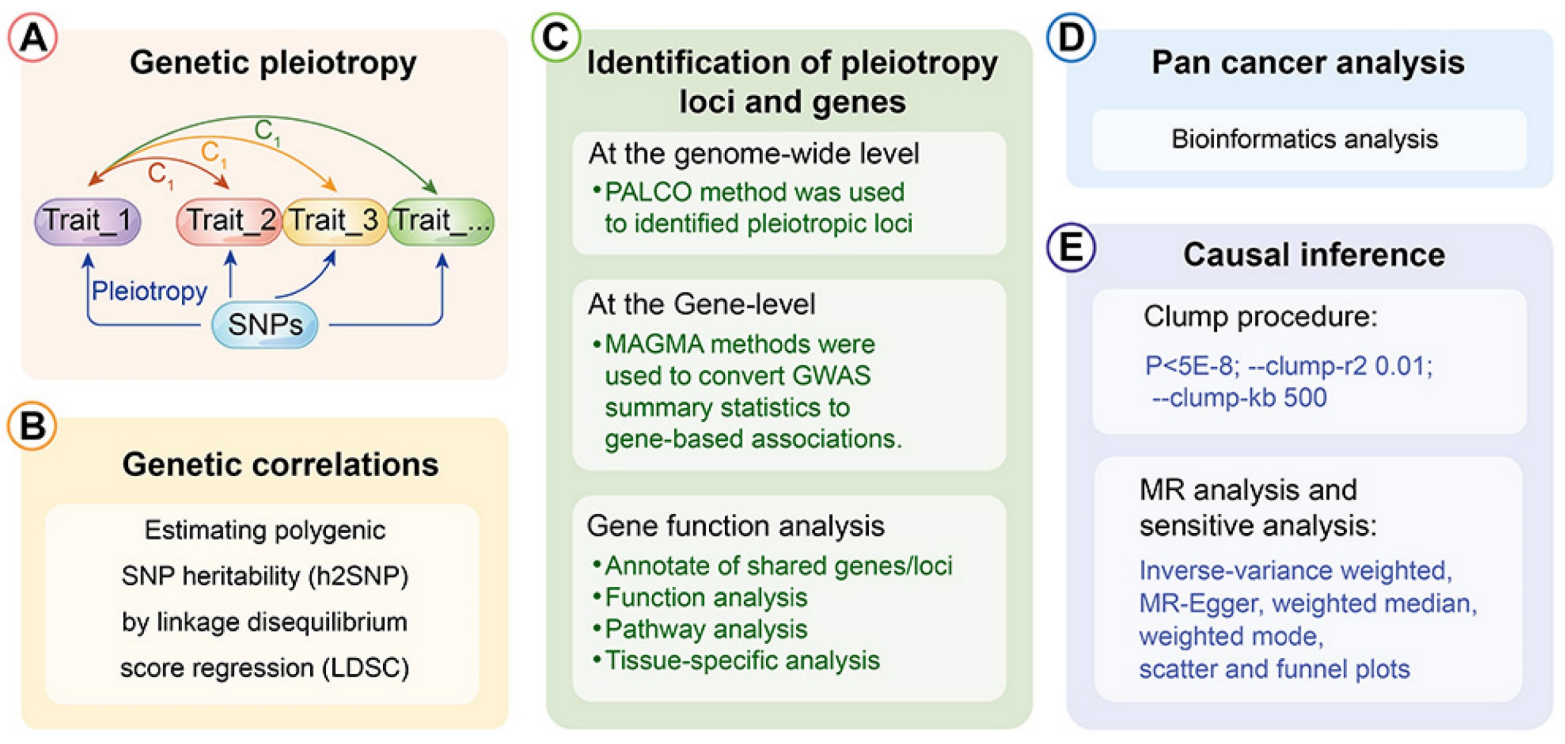
The analytical workflow's schematic overview.

The depiction of the schematic overview of the analytical workflow was shown** (Fig. 1)**. To investigate the relationship between esophageal cancer and lung cancer, we initially assessed their genetic correlation. Through genetic correlation analysis, we found a significant genetic correlation between lung cancer and esophageal cancer, both in LDSC (rg = 0.234, P = 0.001) and in HDL (rg = 0.180, P = 0.032) **(Additional file: Table S1)**.

### 3.2 Estimation of pleiotropic enrichment

**Figure 2:**
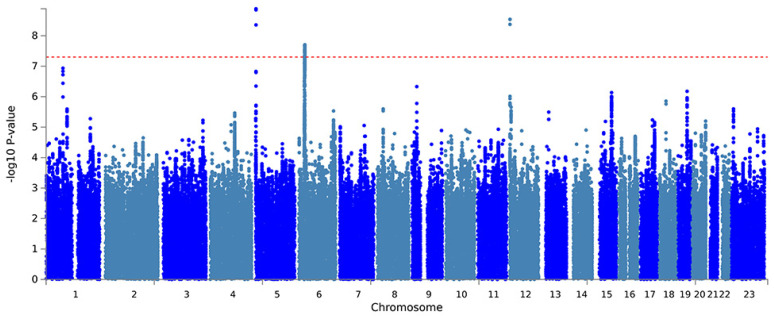
Manhattan plot showing pleiotropic loci between lung cancer and esophageal cancer.

**Figure 3:**
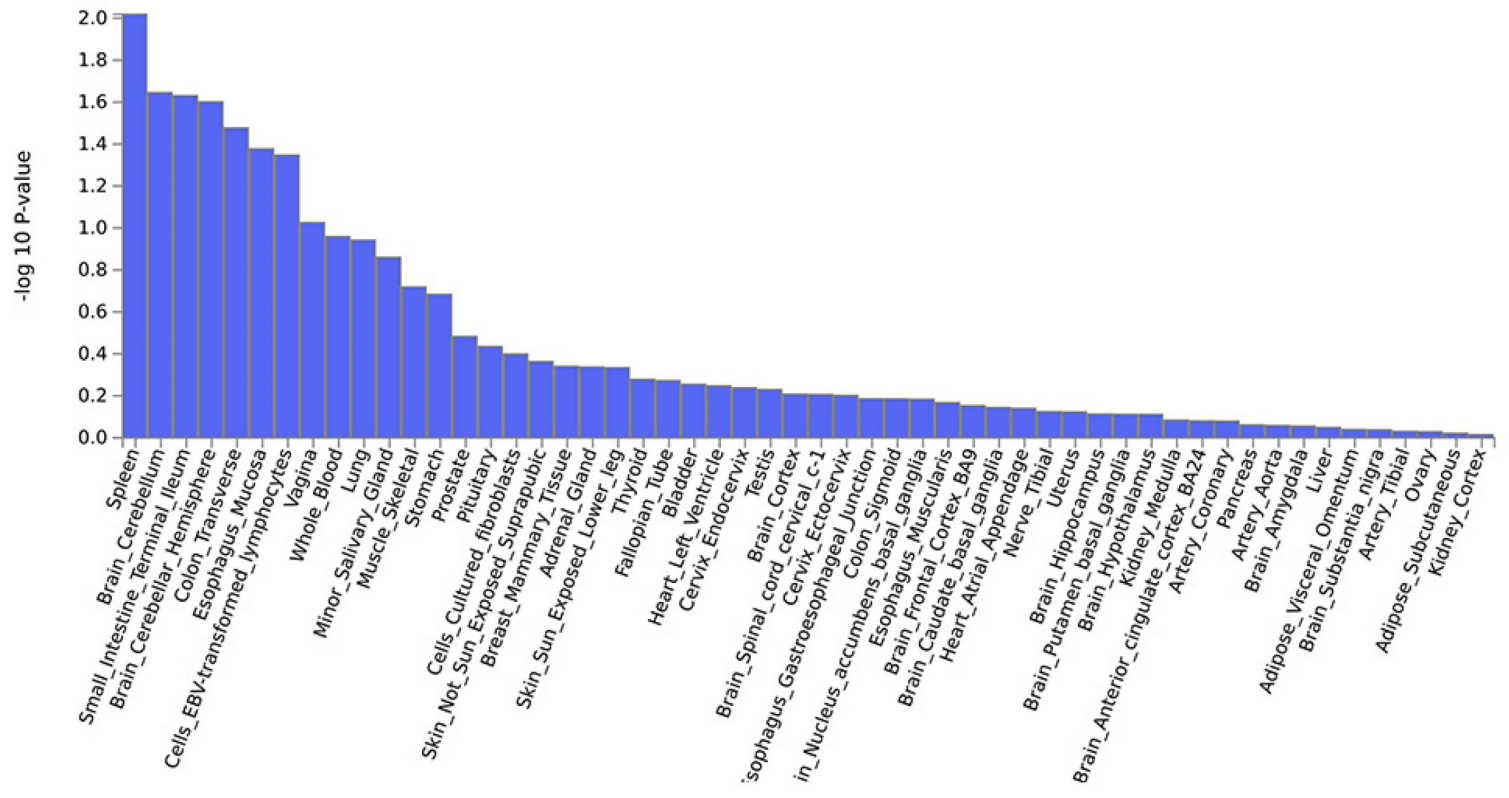
Genome-wide pleiotropy-based MAGMA tissue-specific analysis.

Further, a PLACO analysis was conducted on both diseases, and the Manhattan plot is displayed **(Fig. 2)**. The identified pleiotropic loci are presented, revealing three pleiotropic genes: PGBD1, ZNF323, and WNK1 **(Table 1)**. The QQ plot did not show signs of genomic inflation **(Additional file: Fig. S1)**. The presentation included essential details for each genome-wide risk locus, encompassing the risk locus size, SNP count, mapped gene count, and the number of genes within the locus **(Additional file: Fig. S2)**. The functional impact of the pleiotropic SNPs on genes is illustrated **(Additional file: Fig. S3)**. Additionally, regional plots for each risk locus can be found **(Additional file: Fig. S4-S6)**. Multiple-effect results were subjected to gene-set enrichment analysis using MAGMA, which revealed the top 10 significantly enriched gene sets **(Additional file: Table S2)**. These sets encompassed pathways such as breast cancer 5p15 amplicon, DNA metabolic process, reactome highly calcium permeable postsynaptic nicotinic acetylcholine receptors, EHMT2 targets up, reactome highly calcium permeable nicotinic acetylcholine receptors, regulation of xenophagy, PPARA pathway, PTEN pathway, RNA binding and genomic pathway. Previous literature has provided substantial evidence linking these significantly enriched pathways to cancer initiation and progression, as observed in bladder cancer^38^, gastric cancer^39^, cervical cancer^40^, lung cancer^41^-^43^, and others associated with the amplification or alteration of 5p15; the close association between the PPARA pathway and various cancers^44^-^46^; as well as the role of the PTEN pathway in tumorigenesis^47^-^49^. Tissue-specific MAGMA analysis indicated that the spleen exhibited the highest enrichment evidence for both diseases, suggesting a strong immune correlation **(Fig. 3 and Additional file: Table S3)**. It is essential to note that this section of MAGMA gene-set and tissue-specific analysis utilized the complete distribution of SNP p-values for examination.

In genetic studies, Single Nucleotide Polymorphisms (SNPs) are common genomic variations. Among them, the "lead SNP" refers to the SNP that exhibits the strongest association in genetic association analyses. The role of the lead SNP is to aid in determining the correlation between specific genes or genomic regions and target traits or diseases in genetic association research. It can be considered a representative SNP and serves as a marker or reference point for other related SNPs. By utilizing the positional information of the lead SNP, we identified genes associated with these pleiotropic risk loci **(Table 1)**. Further analysis using MAGMA identified 9 pleiotropic genes: HIST1H1B, HIST1H4L, HIST1H2BL, OR2B2, HIST1H2BN, HIST1H2AL, RAD52, HIST1H2AJ, and HYKK **(Additional file: Fig. S7 and Table S4)**. No genomic inflation was observed in the QQ plots, confirming the reliability of the results **(Additional file: Fig. S8)**. The expression of pleiotropic genes in various tissues, including the thyroid, blood vessels, brain, cervix, tibial nerve, and ovary, showed differential expression **(Additional file: Fig. S9)**. Pathway analysis reveals enrichment in pathways related to protein DNA complex subunit organization, chromatin assembly, nucleosome organization, and DNA packaging, among others **(Additional file: Fig. S10-S13)**.

### 3.3 eQTL analysis

The eQTL analysis is a commonly used multi-omics integration analysis method that allows us to associate changes in gene expression levels with genotypes. It helps reveal the physiological and biochemical processes of living systems, discover genetic factors leading to certain diseases, and identify biological pathways affected by them. Therefore, we conducted expression quantitative trait locus (eQTL) analysis, which is a method employed to investigate the associations between genotypes and phenotypes^50^. This approach delves into how genetic variations influence gene expression levels by analyzing the relationship between single nucleotide polymorphisms (SNPs) and gene expression. In this current study, we further utilized eQTL information from esophageal tissue, lung tissue, and whole-blood data to identify eQTL genes associated with these pleiotropic risk loci **(Additional file: Table S5)**. A total of 26 genes, including TERT, HIST1H2BN, ZSCAN23, OR2H2, SCAND3, PRSS16, ZSCAN31, ZNF391, ZSCAN12, ZKSCAN3, ZNF322, PGBD1, ZKSCAN4, ZNF184, NKAPL, ZNF165, ZNF311, RAD52, ZSCAN9, BTN3A2, ABT1, ZKSCAN8, GABBR1, HIST1H2BK, CLPTM1L, and TRIM27, were identified. The expression patterns of these pleiotropic eQTL genes are presented in different tissues **(Additional file: Fig. S14)**. Notably, these genes exhibited significant enrichment in the uterus, brain, and skeletal muscle tissues **(Additional file: Fig. S15)**. Pathway enrichment analysis involves six immune-related gene sets (UNSTIM VS 2H LPS AND R848 DC UP, CSF1 VS CSF1 IFNG IN MAC DN, HEALTHY VS TYPE 1 DIABETES PBMC 4MONTH POST DX DN, WT VS TCF1 KO DN3 THYMOCYTE UP, UNSTIM VS ANTI IGM AND CD40 STIM 6H FOLLICULAR BCELL DN, CD8A DC VS NK CELL MOUSE 3H POST POLYIC INJ DN) **(Additional file: Fig. S16 and Table S6)**. Furthermore, the protein-protein interaction (PPI) results **(Additional file: Fig. S17)** include genes such as PGBD1, ZKSCAN3, ZKSCAN4, ZKSCAN8, and ZKSCAN23. This comprehensive analysis allows us to gain a deeper understanding of the interplay between genotypes and phenotypes, shedding light on the regulatory mechanisms of gene expression in different tissues.

### 3.4 Pan cancer analysis

**Figure 4:**
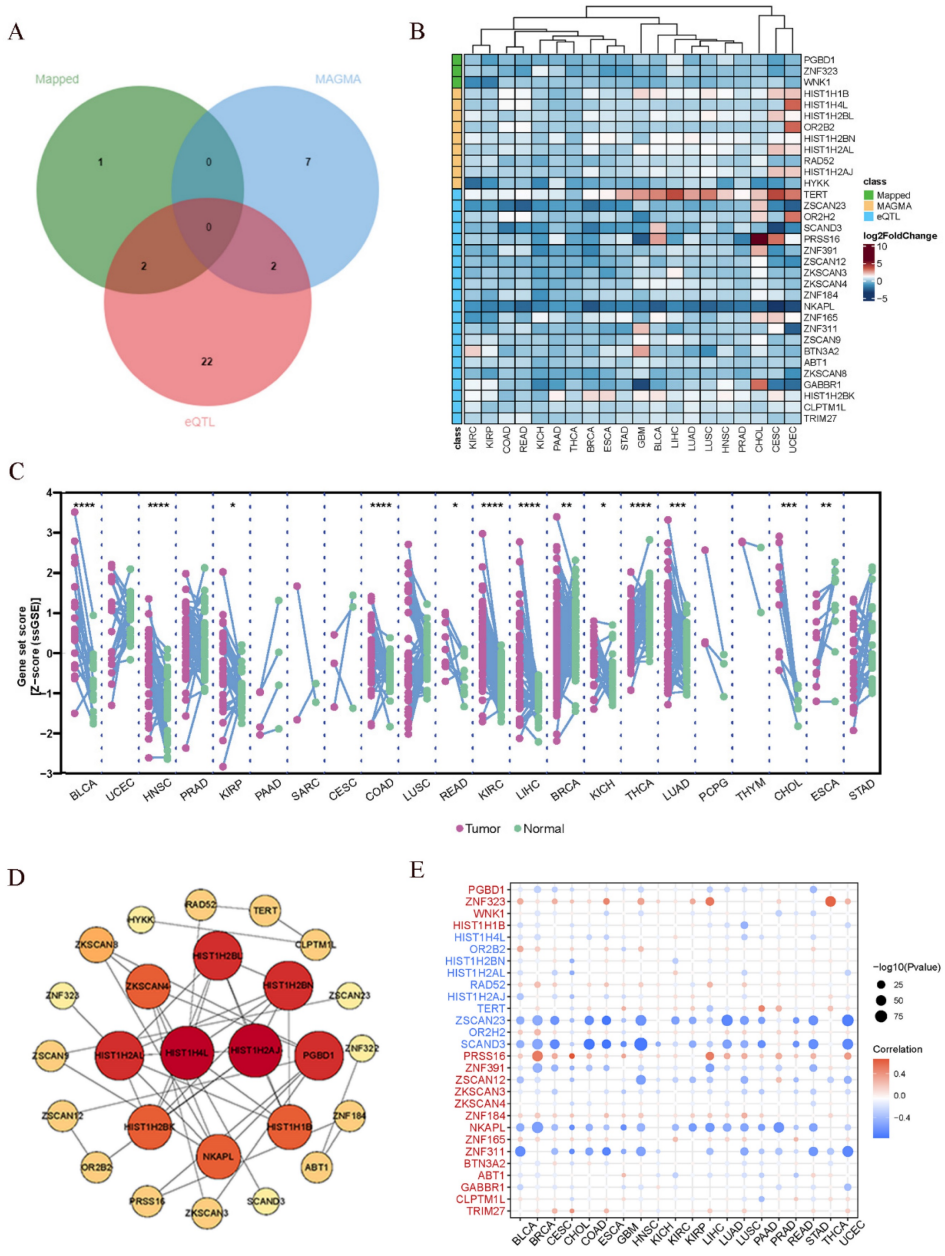
Pan-cancer analysis. **(A)**Venn diagram depicting the distribution of genes from three different sources. **(B)** Gene Expression Across 17 Types of Cancer. The heat map shows the fold changes, with red representing up-regulated genes, and blue representing down-regulated genes. **(C)** Expression landscape of genes in human cancer. Y-axis representing gene set score, which was calculated by ssGSEA based on the gene expression in the TCGA. In paired samples grouped by cancer from the TCGA. Each point representing one sample. p-values are based on two-tailed Student t-test. **(D)** PPI network showing the interactions of hub genes. **(E)** Pearson's correlation of genes between transcriptional expression and promoter methylation. Red and blue represent positive and negative correlations, respectively.

In the preceding analysis, we obtained 3 Mapped genes, namely PGBD1, ZNF323, and WNK1, through PLACO analysis. Additionally, MAGMA analysis revealed nine genes, including HIST1H1B, HIST1H4L, HIST1H2BL, OR2B2, HIST1H2BN, HIST1H2AL, RAD52, HIST1H2AJ, and HYKK. Furthermore, eQTL analysis identified a total of 26 genes, which encompassed TERT, ZKSCAN3, NKAPL, ZNF165, RAD52, BTN3A2, GABBR1, HIST1H2BK, CLPTM1L, and TRIM27. The combined results from these three analytical approaches yielded 38 genes. After eliminating duplicate genes (note: PGBD1 and ZNF323 were replicated from PLACO and eQTL analysis, while HIST1H2BN and RAD52 were duplicated in MAGMA and eQTL analysis; ZNF323 is also recognized as ZSCAN31), the final count of unique genes was reduced to 34. The origin and Venn diagram representation of these genes are depicted** (Fig. 4A)**. Subsequently, we performed an investigation of the chromosomal locations of these genes in the human genome. These genes were distributed across chromosomes 5, 6, 12, and 15, with a notable concentration of twenty-nine genes on chromosome 6, confined to a specific segment **(**Chromosomal Start and End Positions: 26365387…29556745)** (Additional file: Fig. S18 and Table S7)**. Extensive research has linked chromosome 6 to tumorigenesis. For instance, numerous chromosomal aberrations are frequently observed in thymoma patients^51^. Similarly, colorectal cancer patients exhibit imbalanced allelic frequencies of the tumor necrosis factor-alpha gene and chromosome 6 allelic genes^52^. Furthermore, various gene alterations on chromosome 6 have been implicated in the promotion and progression of breast cancer^53^. Collectively, these pieces of evidence suggest the significance of this chromosomal region in the coordinated regulation of esophageal and lung cancer development.

In addition to the positional information, we further explored whether these genes have similar functional or epigenetic connections. The 34 genes obtained from the previous analysis are considered to be involved in the comorbidity between lung and esophageal cancers. We were curious to investigate whether these genes also exhibit similar comorbidity in other cancers. Hence, we conducted a pan-cancer analysis to explore potential interconnections among them. Firstly, we generated a heatmap from the differential expression analysis of the pan-cancer data **(Fig. 4B)**, which revealed that certain genes exhibit similar expression patterns across various cancer types. For instance, TERT displayed a ubiquitous upregulation, while NKAPL exhibited a universal downregulation. Prior research has suggested that TERT is typically expressed in human germ and stem cells but silenced in differentiated somatic cells. However, it undergoes transcriptional reactivation in up to 90% of human malignancies^54^-^56^. Notably, in thyroid cancer, TERT promoter mutations enhance telomerase activity, leading to immortalization of cancer cells^57^. Additionally, identifying TERT promoter mutations from urine or plasma cell-free DNA (liquid biopsy) can aid in early screening of bladder cancer^58^. In the context of liver cancer, the high methylation level and low expression of NKAPL are associated with poor prognosis^59^. These findings suggest that the identified genes may play crucial roles in various cancers and have potential implications in cancer diagnostics and treatment strategies. The pan-cancer analysis has unveiled shared expression patterns, highlighting the importance of exploring these genes as potential therapeutic targets or biomarkers across different cancer types. Further investigations are warranted to elucidate the underlying molecular mechanisms and functional significance of these genes in cancer development and progression.

Next, utilizing the ssGSEA algorithm, we converted the comprehensive expression of each gene into a Z-score and conducted paired differential analysis between tumor and adjacent normal tissues across various cancers in TCGA. Remarkably, significant differences were observed in several cancers, including esophageal cancer and lung cancer **(Fig. 4C)**. Then we conducted a Protein-Protein Interaction (PPI) network analysis to explore the interplay between proteins and discovered tight interconnections among them **(Fig. 4D)**. This network analysis helped unveil the functional associations and mutual interactions between proteins, providing valuable insights into their collective behaviors in esophageal and lung cancer. Furthermore, single pathway Gene Set Enrichment Analysis (GSEA) was performed on each KEGG pathway **(Additional file: Fig. S19)**. The DNA repair pathway, E2F signaling pathway, G2M checkpoint signaling pathway, mitotic spindle signaling pathway, and MYC signaling pathway exhibited positive correlations with various types of cancers. These findings provide additional insights into the potential roles of these genes in cancer development and progression. The identification of associations with survival, gene expression differences between tumor and normal tissues, and promoter methylation patterns enhances our understanding of the molecular mechanisms underlying cancer pathogenesis. The enrichment of specific pathways further supports the notion that these genes may be involved in critical cancer-related processes, warranting further investigation for their clinical significance and therapeutic potential. Gene promoter methylation is a common epigenetic event that occurs in the early stages of tumor development and holds immense potential as a diagnostic and prognostic biomarker for cancer^60^. Consequently, we conducted a pan-cancer analysis of gene promoter methylation and observed significant associations between several genes, including ZNF323, ZSCAN23, SCAND3, PRSS16, ZNF391, NKAPL, and ZNF311, and promoter methylation **(Fig. 4E)**.

The pan-cancer analysis looking across different cancer types reveals some interesting connections. Several of the genes we identified as being important in lung and esophageal cancers also seem to play roles in other cancers. For example, the TERT gene shows increased activity across almost all the cancers we looked at. Previous research has shown that TERT activity is increased in many cancer cells and helps the cells divide indefinitely. So our findings suggest this gene could be a useful target for cancer therapies. Another gene called NKAPL shows decreased activity in the cancers we studied. Other research found low NKAPL levels were linked with worse outcomes in liver cancer. Overall, our pan-cancer analysis suggests that some of the genes important in lung and esophageal cancer are also dysregulated in other cancer types. Targeting these shared genes could potentially lead to new diagnostic tests or treatments that apply to multiple cancers. We plan to explore these possibilities in future studies.

### 3.5 Mendelian randomization analysis

**Figure 5:**
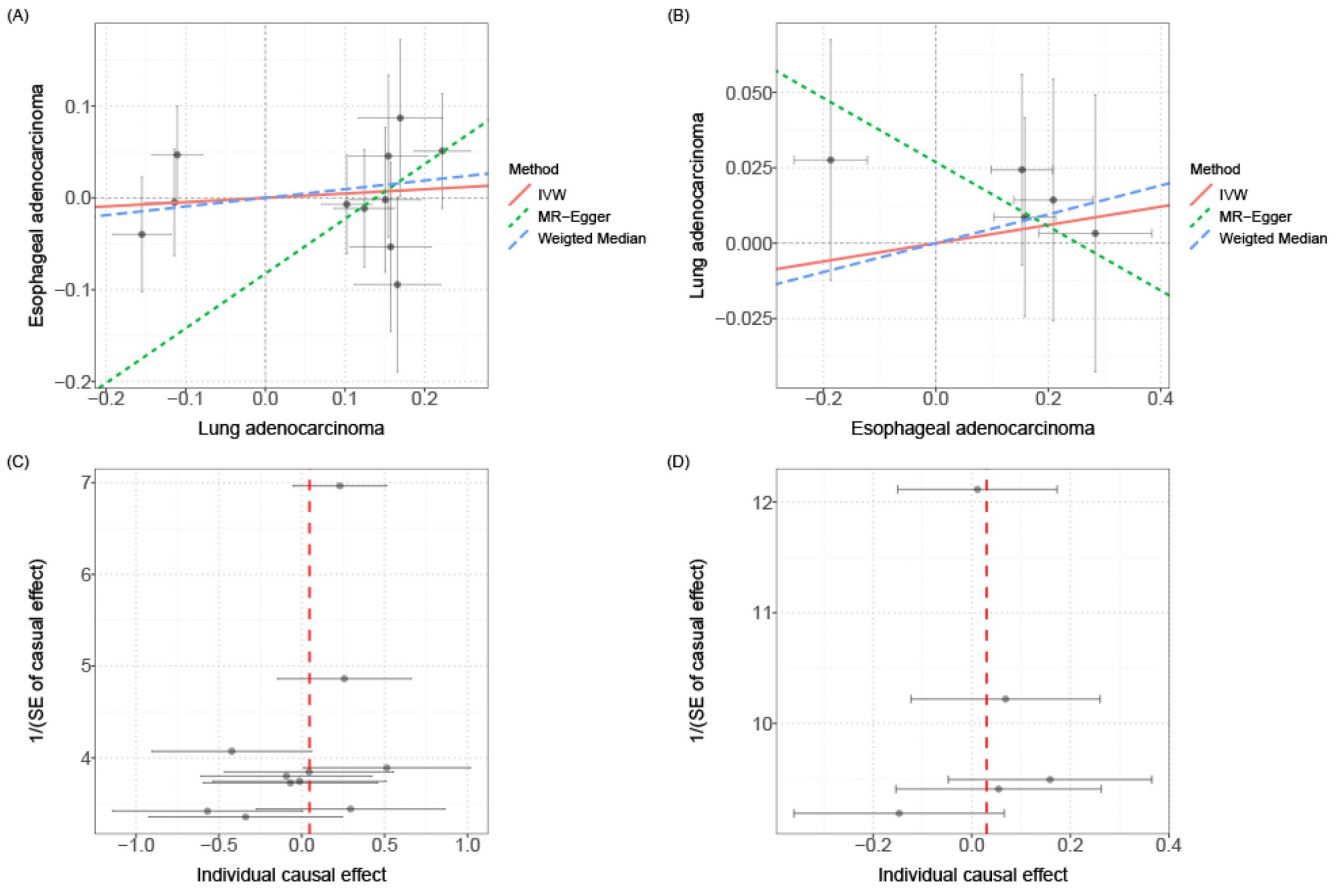
Scatter plot and funnel plot for MR analysis. **(A)** Scatter plot of causal effects of lung cancer on esophageal cancer. **(B)** Scatter plot of causal effects of esophageal cancer on lung cancer. **(C)** Funnel plot of causal effects of lung cancer on esophageal cancer. **(D)** Funnel plot of causal effects of esophageal cancer on lung cancer. The bidirectional MR results do not support any causal association among them.

Mendelian Randomization (MR) is a commonly used causal inference tool that employs single nucleotide polymorphisms (SNPs) associated with the exposure of interest as instruments to study its impact on outcomes^31^. In order to explore whether there exists a causal relationship between esophageal cancer and lung cancer, we conducted causal inference using the two-sample MR approach. However, the results did not support a significant association between the two diseases. The instrumental variables **(Additional file: Table S8)**, sensitivity analysis **(Table 2 and Additional file: Table S9)** and scatter plots and funnel plots **(Fig. 5)** are available for reference. The causal effect analysis of lung cancer on esophageal cancer revealed consistent results across all four MR methods (IVW, MR-Egger, Weighted mode, Weighted median), indicating the absence of a causal effect of lung cancer on esophageal cancer. The heterogeneity test yielded a P-value of 0.353, suggesting no significant heterogeneity, and the MR-Egger intercept had a P-value greater than 0.05, indicating the absence of horizontal pleiotropy. Scatter plots and funnel plots were employed to rule out the possibility of outlier interference. Similarly, there was no causal effect of esophageal cancer on lung cancer, as all four MR methods (IVW, MR-Egger, Weighted mode, Weighted median) yielded consistent results. The heterogeneity test resulted in a P-value of 0.075, indicating no significant heterogeneity, and the MR-Egger intercept had a P-value greater than 0.05, suggesting the absence of horizontal pleiotropy. Scatter plots and funnel plots were utilized to exclude the interference of outliers.

Overall, while our MR analysis results suggest a lack of clear causal relationship between lung cancer and esophageal cancer, this helps focus on other potential mechanisms of carcinogenesis. Furthermore, given the limited sample size, the possibility of a weaker causal effect cannot be completely ruled out. Larger sample datasets will need to be tested in future studies to arrive at more robust causal conclusions.

## 4. Discussion

Our study findings hold significant implications from both statistical and scientific perspectives. Firstly, the extensive genetic overlap among tumors contributes to the identification of potential genetic connections that might remain concealed in single-phenotype studies. Furthermore, harnessing phenotypic correlations across tumor characteristics significantly enhances the statistical power and predictive accuracy in joint analyses.

This signifies that the application of efficient pleiotropy-informed statistical methods to explore existing and future tumor datasets can yield more fruitful returns. Secondly, a comprehensive understanding of the shared genetic architecture of tumors is crucial for the development of novel gene-based therapeutic strategies. It is highly plausible that targeted treatment designed for one disease may exert broader therapeutic effects across other tumors, potentially benefiting a larger cohort of patients. Thirdly, within the domain of pleiotropic associations, we underscore the phenomenon known as "antagonistic effects," wherein a specific gene may exhibit robust associations with multiple tumors, but the direction of its genetic effects could be opposite in different tumor types. This finding holds particular importance in the discovery of molecular targets, especially for genome-editing techniques like the CRISPR-CAS system, as unforeseen genetic and consequent phenotypic side effects may arise. Lastly, the use of bioinformatics approaches helps reveal potential associations between diseases^61^ [62]and the exploration of causal relationships between distinct tumors offers valuable insights into the development of preventive and therapeutic strategies in clinical settings.

Our findings may have important clinical implications. The identified pleiotropic genes and pathways could represent novel targets for developing preventive and therapeutic strategies that concurrently address multiple cancer types. For example, the ubiquitously amplified TERT gene implicates telomerase inhibition as a possible broad-spectrum cancer treatment approach. Additionally, the proposed prognostic value of genes like NKAPL warrants further validation as clinical biomarkers to refine risk stratification, screening protocols, and treatment decisions. Our discovery of shared genetic mechanisms between lung and esophageal cancers provides a foundation for exploring combinatorial therapies, which may confer synergistic effects against both malignancies. Moreover, the elucidation of causal relationships aids in developing interventions that target key driver phenotypes to mitigate risk. Future drug development efforts could focus on agents that modulate the shared pathways uncovered in our study to generate wider anti-cancer benefits.

However, it is important to acknowledge several limitations in our study. Firstly, our research primarily relied on GWAS data and statistical analyses, which means that we cannot directly ascertain the specific biological mechanisms by which genetic variations influence cancer. Further experimental research and functional validation are necessary to elucidate these mechanisms. Secondly, our study focused on the genetic relationships between lung cancer and esophageal cancer, which may limit the generalization of our findings to other types of cancers and genetic diseases. And there is currently no way to correct for covariates and other heterogeneities in wo-sample MR analysis. Future research could extend to include a broader spectrum of cancer types and diseases. Additionally, all the data used in our study were from European populations, and as such, our conclusions may not be entirely applicable to other ethnicities. Lastly, the data utilized were summary data, which precludes further population stratification analyses (such as different genders or age groups).

## 5. Conclusion

The identified pleiotropic genes and pathways between lung and esophageal cancers represent potential novel targets for developing preventive and therapeutic strategies that concurrently address multiple cancer types. For instance, the ubiquitously amplified TERT gene implicates telomerase inhibition as a possible broad-spectrum cancer treatment approach. Additionally, the proposed prognostic value of genes like NKAPL warrants further validation as clinical biomarkers to refine risk stratification, screening protocols, and treatment decisions. Our discovery of shared genetic mechanisms between lung and esophageal cancers lays the foundation for exploring combinatorial therapies, which may confer synergistic effects against both malignancies. Moreover, the elucidation of causal relationships aids in developing interventions that target key driver phenotypes to mitigate risk. Future drug development efforts could concentrate on agents that modulate the shared pathways uncovered in our study to generate wider anti-cancer benefits.

In conclusion, through the design and methods employed in this study, we have gained preliminary insights into the genetic relationship between lung cancer and esophageal cancer. These research findings hold the potential to contribute to a deeper understanding of the underlying mechanisms of these two cancers, aid in the development of personalized treatment approaches, and provide crucial scientific evidence for early prevention efforts. Future research endeavors should further explore the functional roles and mechanisms of these genetic risk factors and validate their clinical implications through clinical practice.

## Supplementary Material

Supplementary figures and tables.

## Figures and Tables

**Table 1:** Information on the identified 3 pleiotropic loci.

Genomic Locus	uniqID	chr	start	end	LeadSNPs	P-value	Mapped genes
1	5:1307910:A:G	5	1270983	1374233	rs186730568	1.30E-09	
2	6:28290328:G:T	6	27300310	29290172	rs56075693	1.98E-08	PGBD1, ZNF323
3	12:1002857:C:T	12	861374	1077894	rs4619206	2.89E-09	WNK1

**Table 2:** MR analysis results.

Exposure	Outcome	Methods	Estimate	*P*	Heterogeneity test	
					Estimate	*P*
Lung cancer	Esophageal cancer	IVW (fixed)	1.031 (0.945, 1.124)	0.493	4.412	0.353
		MR-Egger (slope)	0.899 (0.438, 1.846)	0.670		
		MR-Egger (intercept)	0.027 (-0.111, 0.165)	0.579		
		Weighted mode	1.043 (0.907, 1.2)	0.555		
		Weighted median	1.049 (0.937, 1.174)	0.405		
Esophageal cancer	Lung cancer	IVW (fixed)	1.048 (0.911, 1.205)	0.513	16.977	0.075
		MR-Egger (slope)	1.192 (0.916, 1.553)	0.192		
		MR-Egger (intercept)	1.099 (0.901, 1.34)	0.352		
		Weighted mode	1.815 (0.823, 4)	0.122		
		Weighted median	-0.083 (-0.198, 0.033)	0.139		
