# Supplementary material for: Illuminating Shared Genetic Associations Between Oesophageal Carcinoma and Pulmonary Carcinoma Risk
Source: J Cancer. 2024 Mar 4;15(8):2412–23. doi: 10.7150/jca.92899 (PMC10937272; doi:10.7150/jca.92899)

**Fig S1.** QQ plot for pleiotropy analysis.


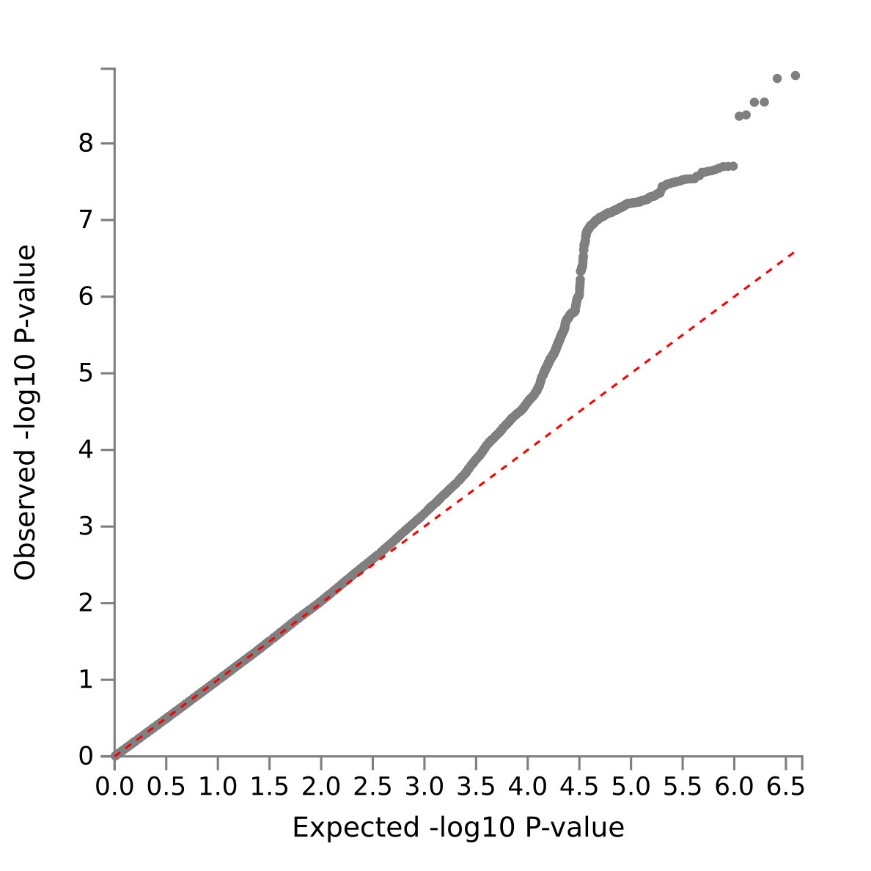


**Fig S2.** Summary of basic information for each genome risk locus.


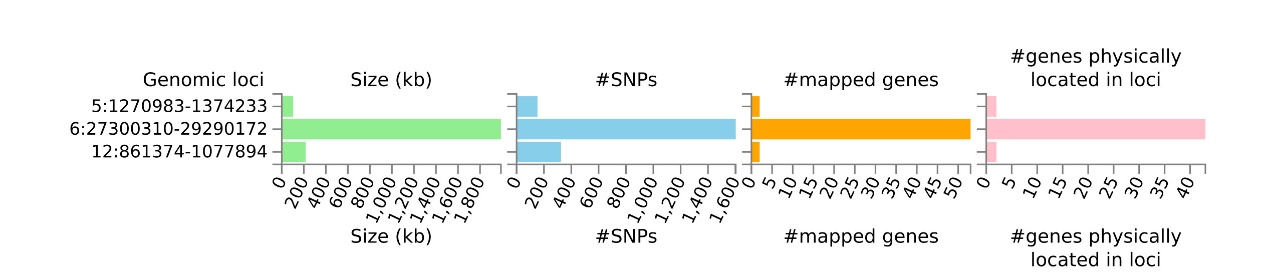


**Fig S3.** Functional impact of pleiotropic SNPs on genes.


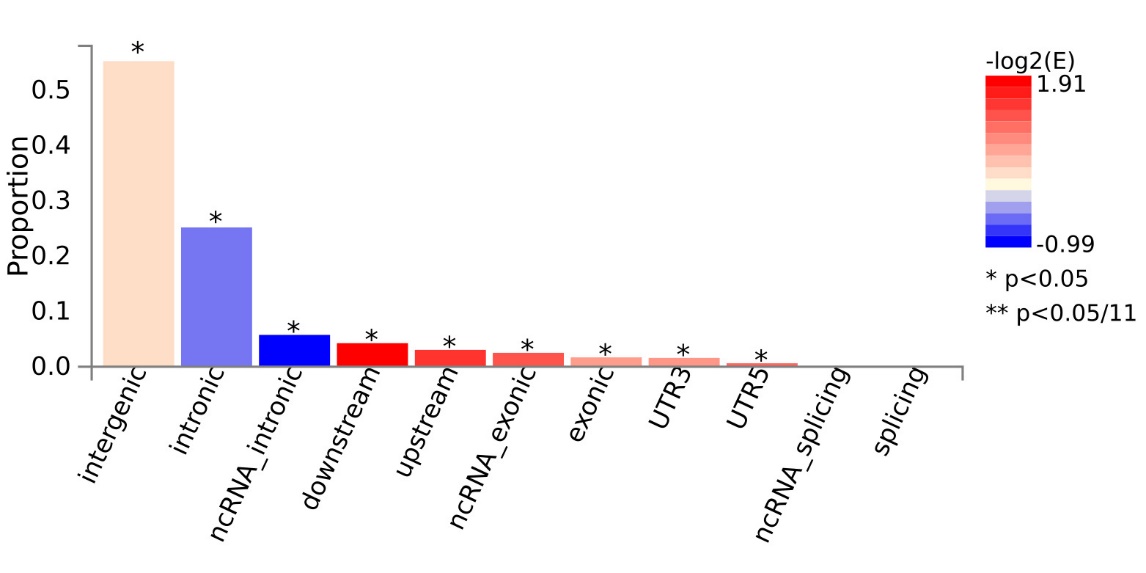


**Fig S4.** Regional plot for locus (5:1307910:A:G).


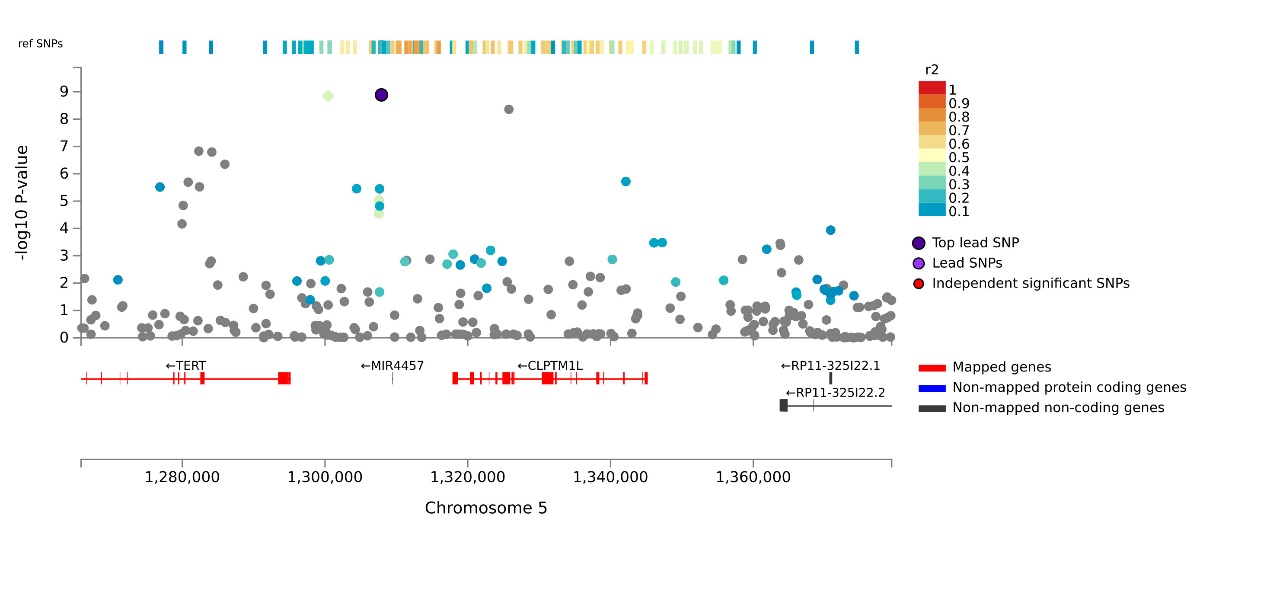


**Fig S5.** Regional plot for locus (6:28290328:G:T).


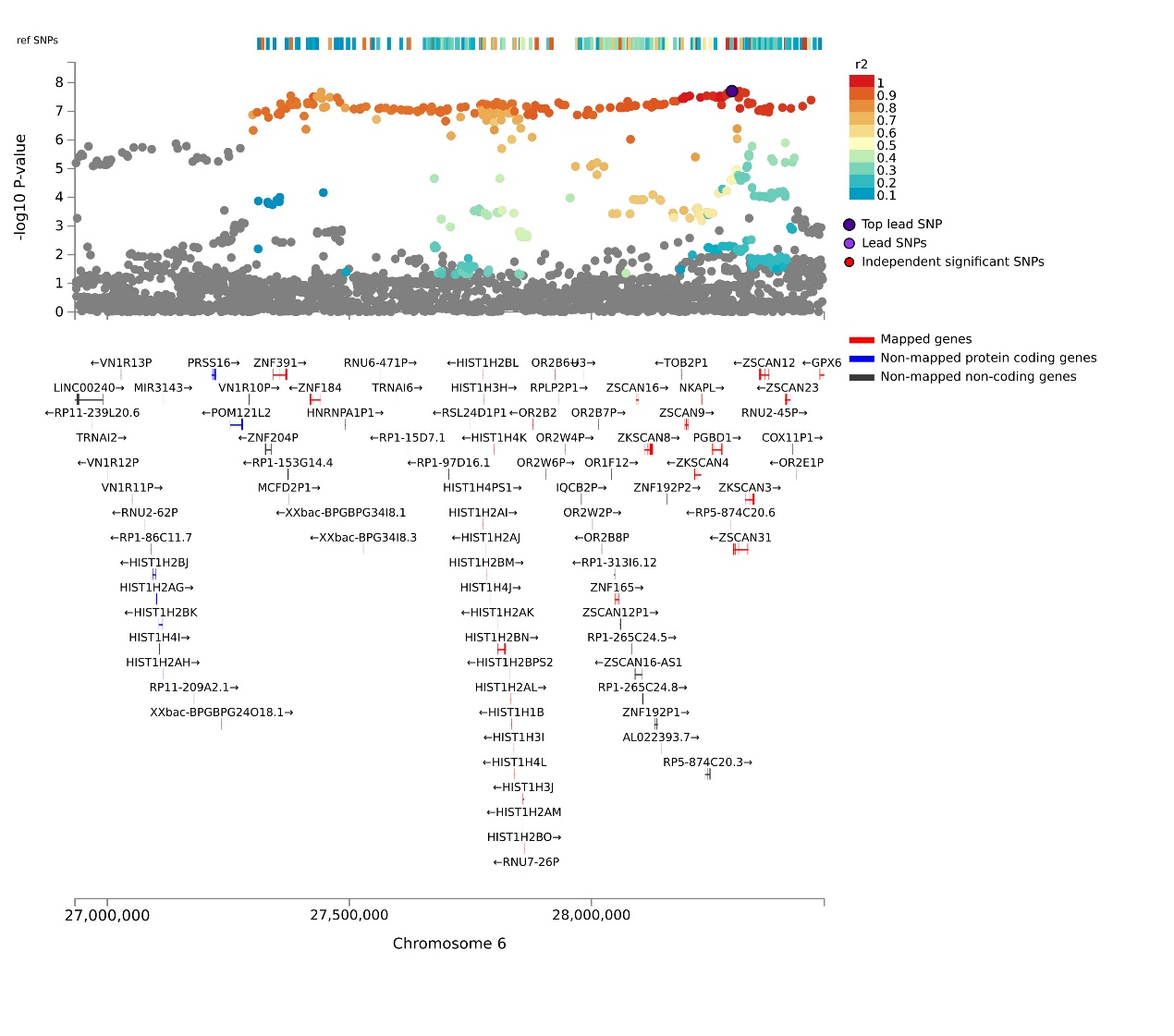


**Fig S6.** Regional plot for locus (12:1002857:C:T).


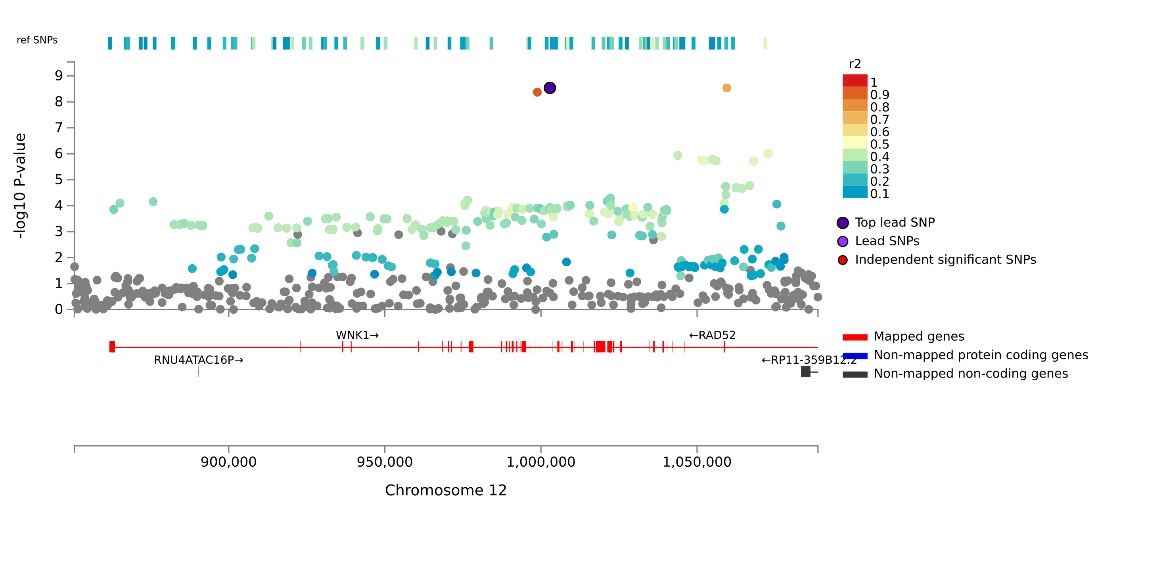
Figure **Fig S7.** Manhattan plot for MAGMA gene analysis.


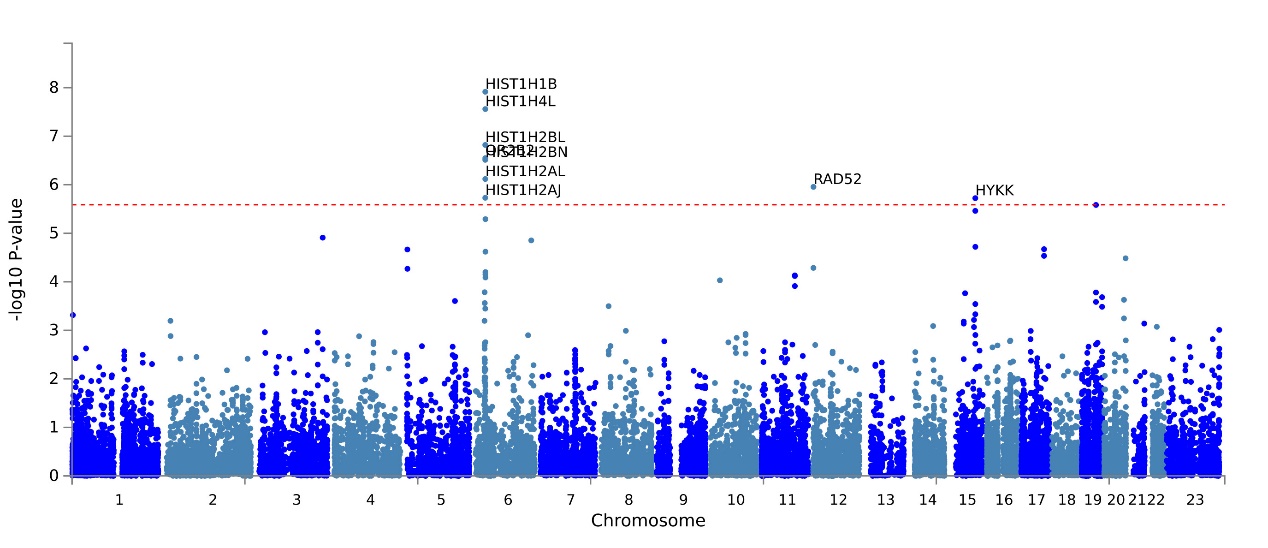


**Fig S8.** QQ plot for MAGMA gene analysis.


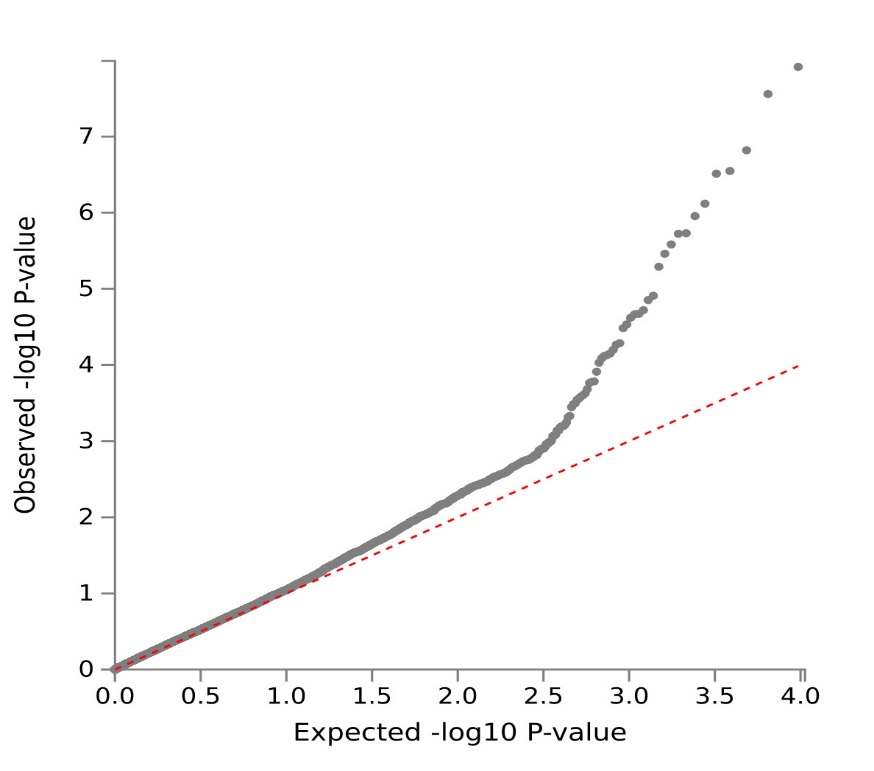


**Fig S9.** Expression profile of pleiotropic MAGMA genes in different tissues.


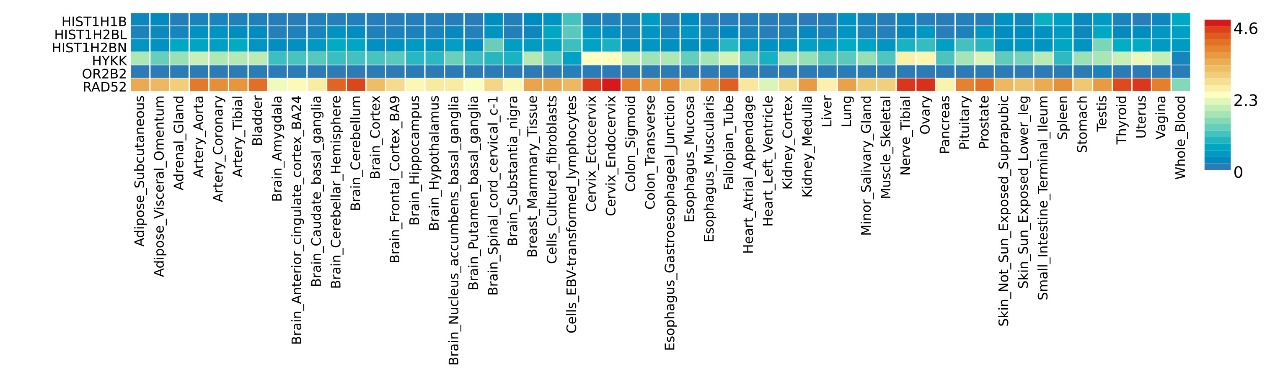


**Fig S10.** Pathway enrichment of pleiotropic MAGMA genes (GO:BP).


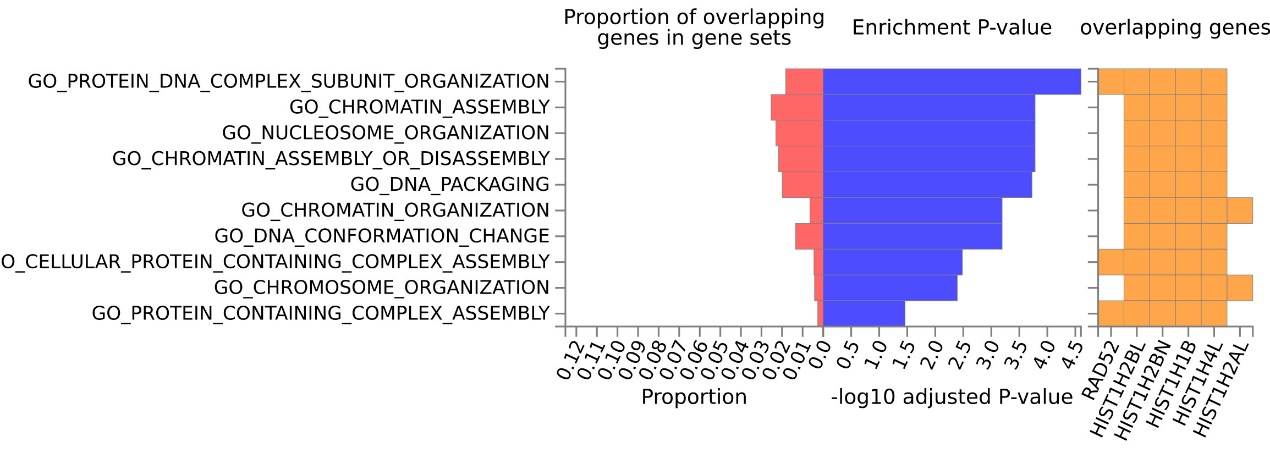


**Fig S11.** Pathway enrichment of pleiotropic MAGMA genes (GO:CC).


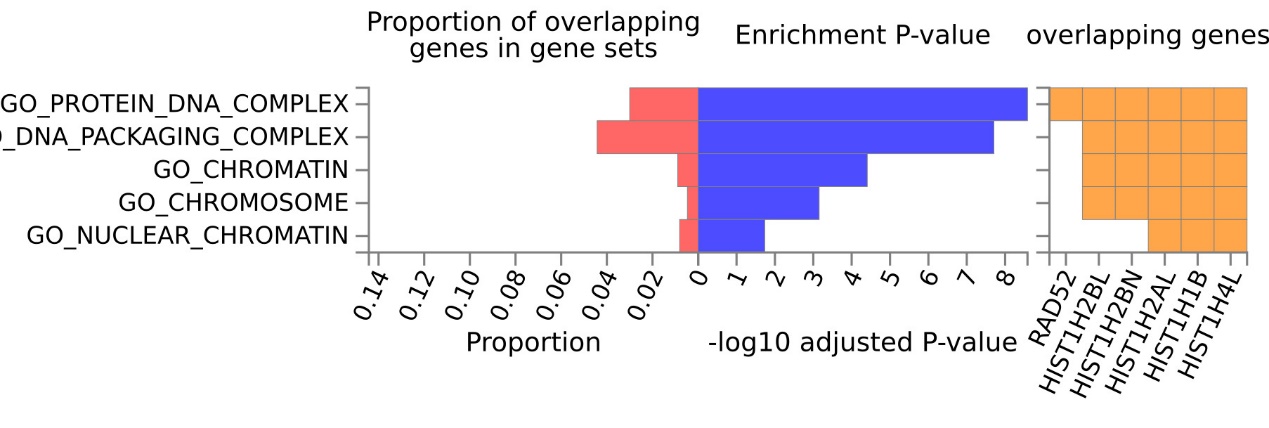


**Fig S12.** Pathway enrichment of pleiotropic MAGMA genes (GO:MF).


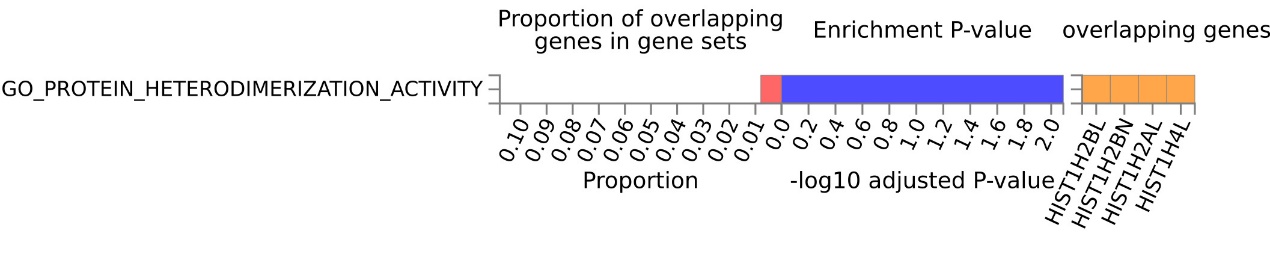


**Fig S13.** Pathway enrichment of pleiotropic MAGMA genes (immune-related pathways).


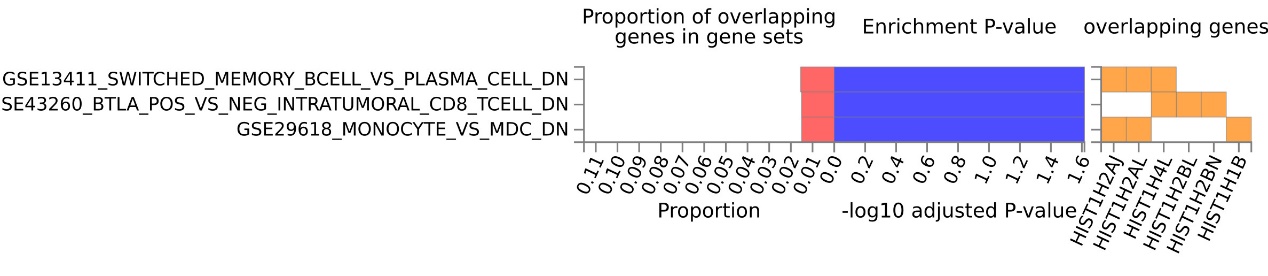


**Fig S14.** Expression profile of pleiotropic eQTL genes in different tissues.


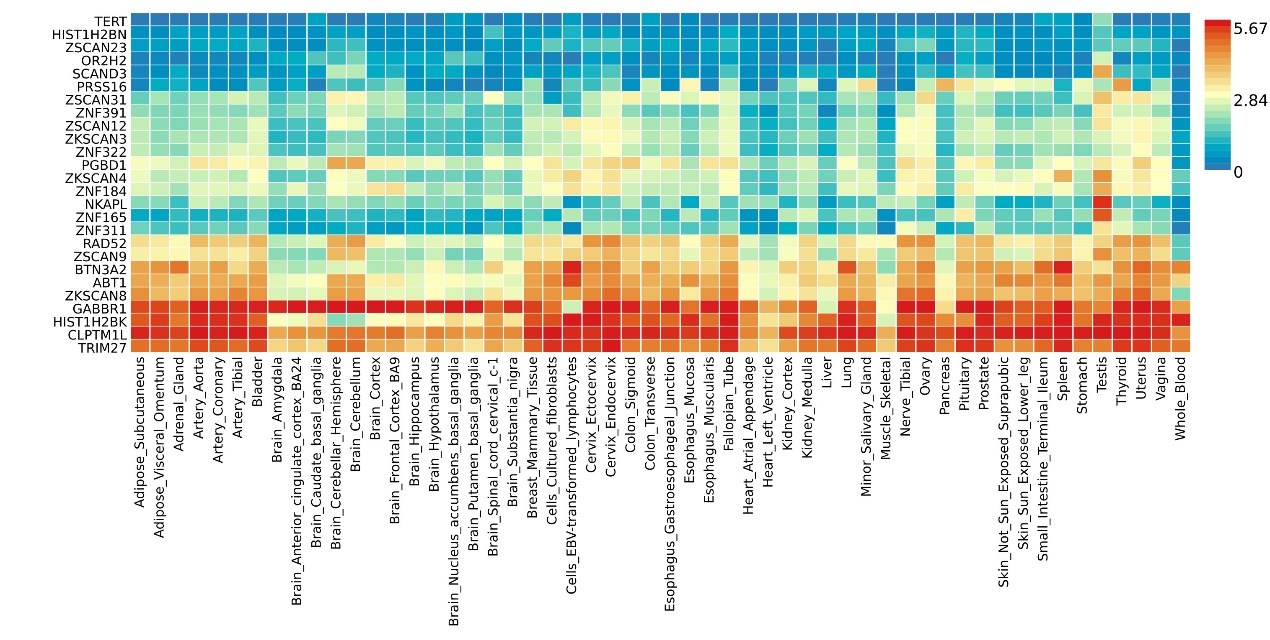


**Fig S15.** Tissue-specific enrichment analysis based on pleiotropic eQTL genes.


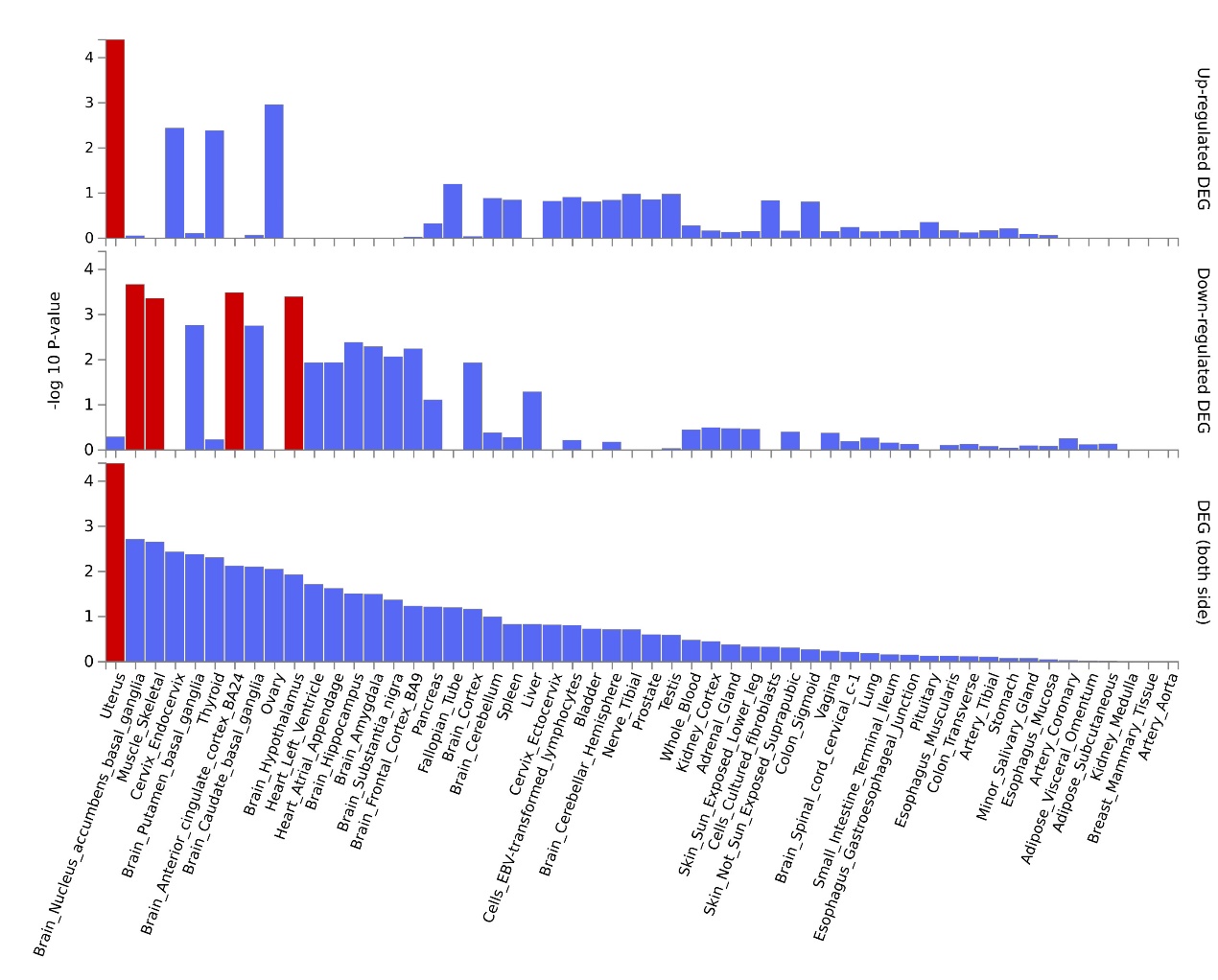


**Fig S16.** Pathway enrichment of pleiotropic eQTL genes.


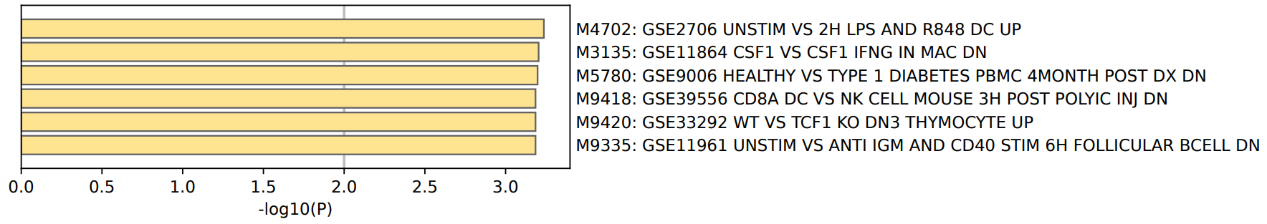


**Fig S17.** Protein-protein interaction (PPI) network analysis of pleiotropic eQTL genes.


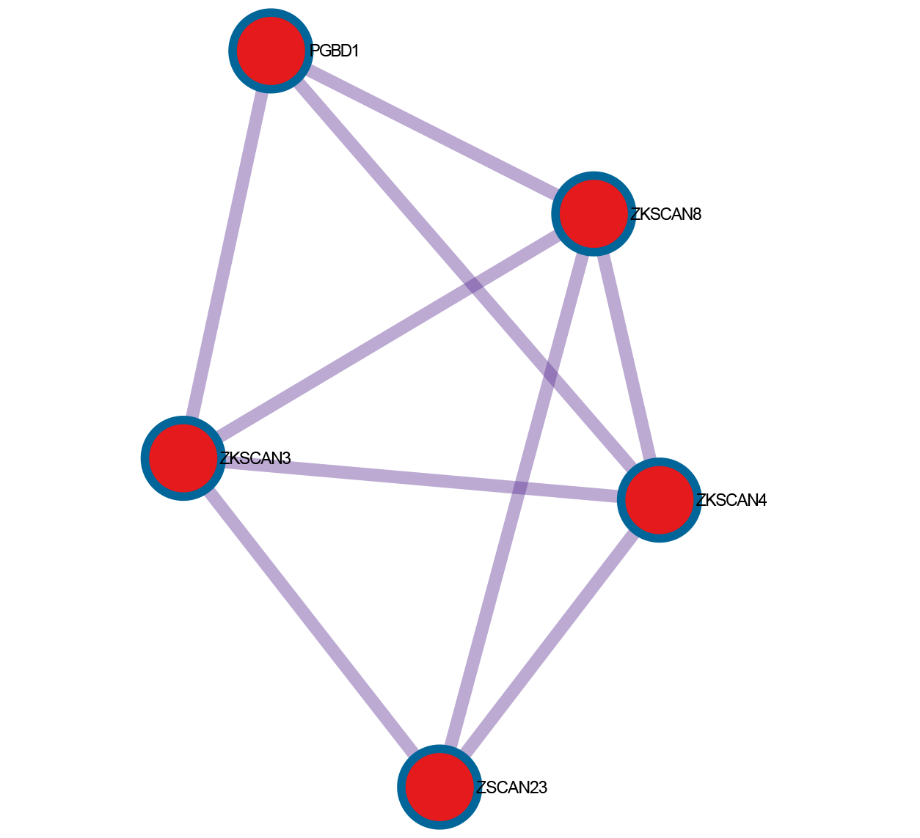


**Fig S18.** Genetic Loci on Chromosomes.

**
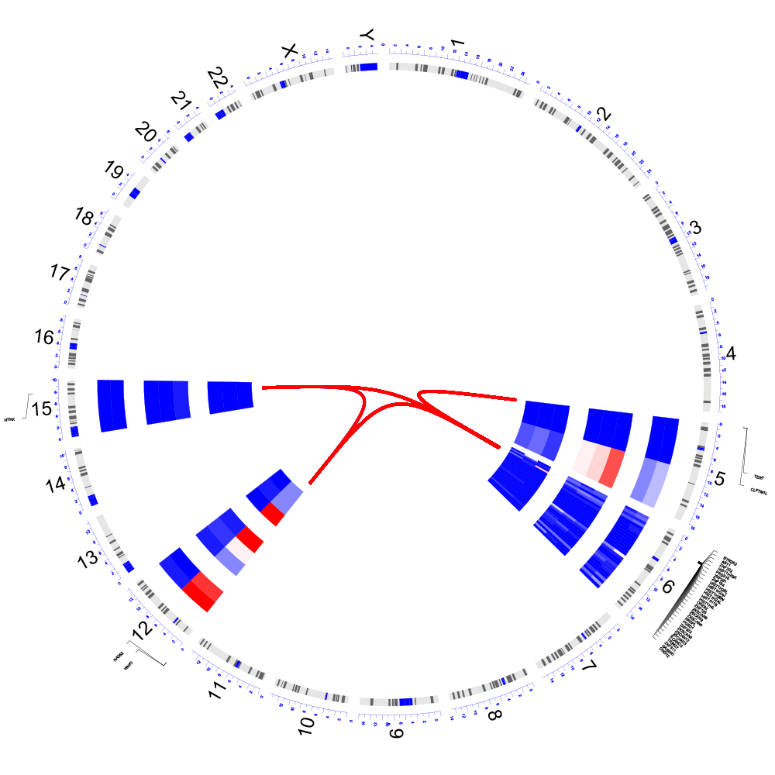
**

**Fig S19.** ssGSEA on each KEGG pathway.


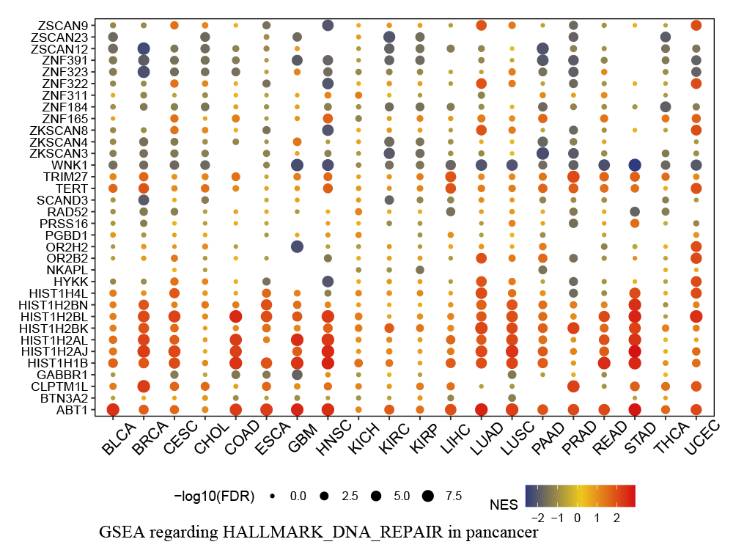

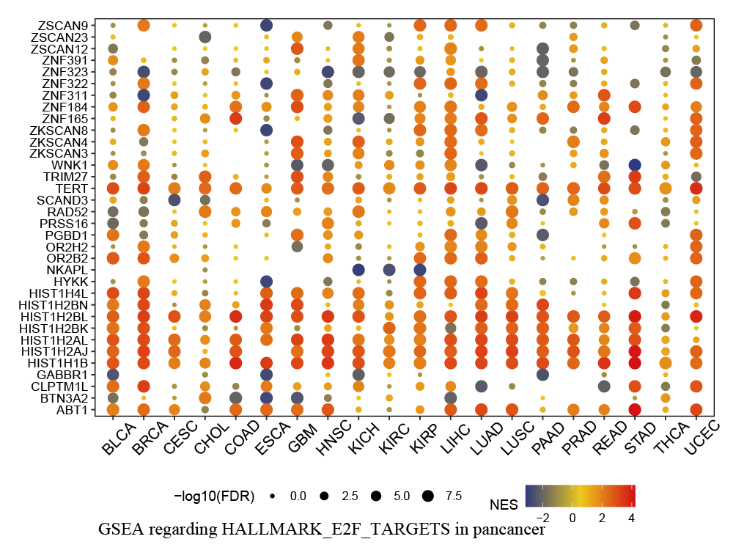

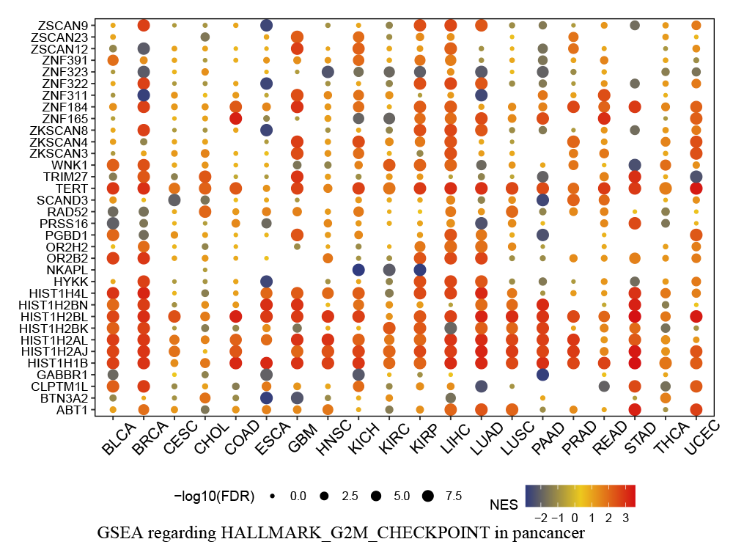

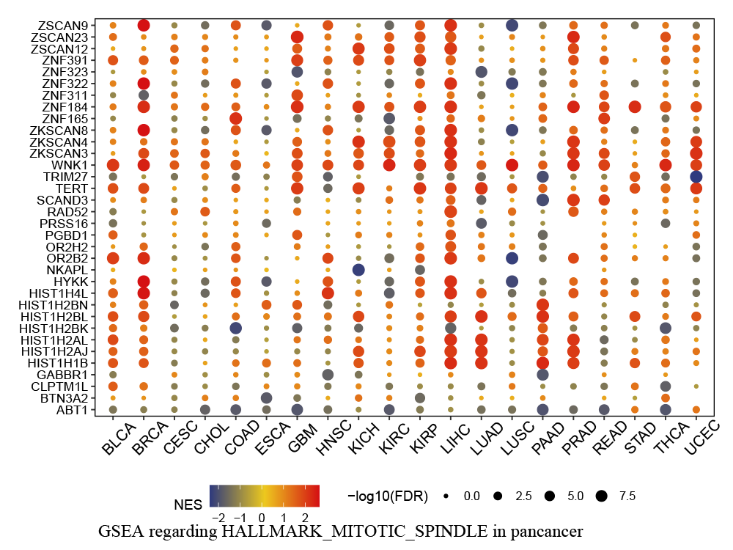

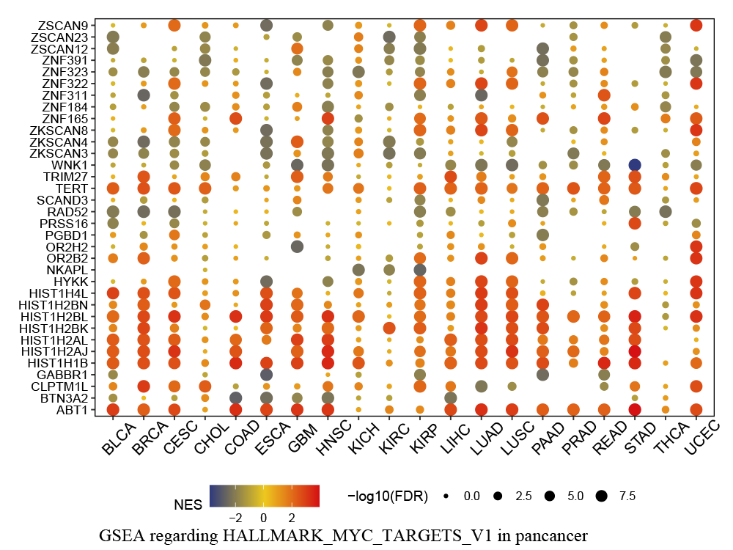

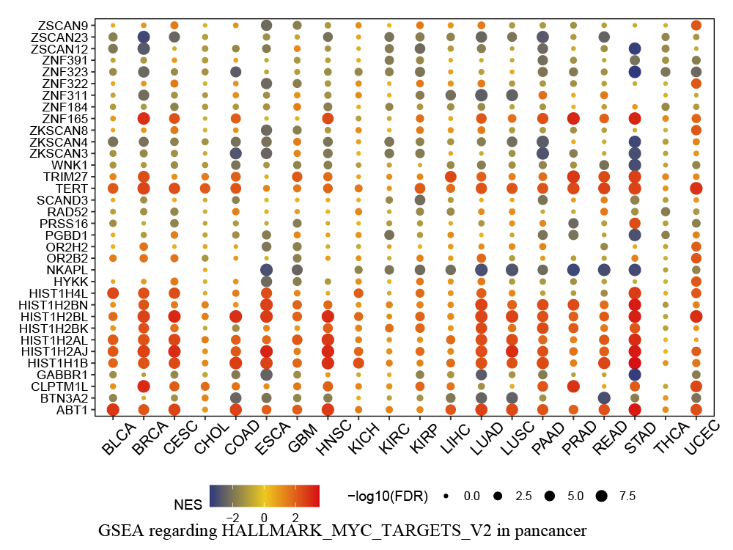

Supplement: Supplementary file 1 — Supplementary figures and tables. [file jcav15p2412s1.zip › FigS1-S19.docx]
